# The CXCR4 antagonist plerixafor (AMD3100) promotes proliferation of Ewing sarcoma cell lines in vitro and activates receptor tyrosine kinase signaling

**DOI:** 10.1186/s12964-018-0233-2

**Published:** 2018-05-18

**Authors:** Philipp Berning, Christiane Schaefer, Dagmar Clemens, Eberhard Korsching, Uta Dirksen, Jenny Potratz

**Affiliations:** 1 Department of Pediatric Hematology and Oncology, University Hospital Münster, Albert-Schweitzer-Campus 1, 48149 Münster, Germany; 2 Division of Hematology and Oncology, Department of Pediatrics III, West German Cancer Centre, University Hospital Essen, Hufelandstraße 55, 45147 Essen, Germany; 3 Institute of Bioinformatics, Westfälische Wilhelms-Universität Münster, 48149 Münster, Germany; 4 Department of General Pediatrics, University Hospital Münster, Albert-Schweitzer-Campus 1, 48149 Münster, Germany; 5 Present address: Department of Medicine A, Hematology, Oncology and Pneumology, University Hospital Münster, Albert-Schweitzer-Campus 1, 48149 Münster, Germany

**Keywords:** CXCR4, Plerixafor, AMD3100, Receptor tyrosine kinases, PDGFRB, SRC, Ewing sarcoma

## Abstract

**Background:**

The CXCR4 receptor antagonist plerixafor (AMD3100) is raising interest as an anti-cancer agent that disrupts the CXCL12-CXCR4 chemokine – receptor interaction between neoplastic cells and their microenvironment in tumor progression and metastasis. Here, we investigated plerixafor for anti-cancer activity in Ewing sarcoma, a rare and aggressive cancer of bone and soft tissues.

**Methods:**

We used a variety of methods such as cell viability and migration assays, flow cytometry, phospho-tyrosine arrays and western blotting to determine plerixafor effects on five characterized Ewing sarcoma cell lines and a low-passage culture in vitro.

**Results:**

Unexpectedly, plerixafor led to an increase in cell viability and proliferation in standard cell growth conditions, and to chemotactic migration towards plerixafor. Exploring potential molecular mechanisms underlying this effect, we found that Ewing sarcoma cell lines divided into classes of high- and low-level CXCR4 surface expression. Proliferative plerixafor responses were observed in both groups, maintained despite significant CXCR4 down-regulation or inhibition of Gαi-protein signal transduction, and involved activation of multiple receptor tyrosine kinases (DDR2, MERTK, MST1R, NTRK1, RET), the most prominent being platelet-derived growth factor receptor beta (PDGFRB). PDGFRB was activated in response to inhibition of the CXCL12-CXCR4 axis by plerixafor and/or pertussis toxin (Gαi-protein inhibitor). Dasatinib, a multi-kinase inhibitor of both PDGFRB and the CXCR4 downstream kinase SRC, counteracted this activation in some but not all cell lines.

**Conclusion:**

These data suggest a feedback interaction between the CXCR4 chemokine receptor and RTK signaling cascades that elicits compensatory cell survival signaling and can shift the net effect of plerixafor towards proliferation. PDGFRB was identified as a candidate driver RTK and potential therapeutic co-target for CXCR4 in Ewing sarcoma. Although as yet limited to in vitro studies, these findings call for further investigation in the cancer – microenvironment interplay in vivo.

**Electronic supplementary material:**

The online version of this article (10.1186/s12964-018-0233-2) contains supplementary material, which is available to authorized users.

## Background

The chemokine network, initially described to mediate homing of immune cells and their directional migration during inflammation, is now gaining interest in the search for therapeutic strategies that target similar interactions of neoplastic cells with the stromal microenvironment in cancer progression (reviewed in [1, 2]). CXCR4 is the chemokine receptor most broadly expressed across normal tissues but also hematologic and solid malignancies (reviewed in [1]), and expression correlates with metastasis and shortened patient survival [3, 4]. Its cognate chemokine ligand CXCL12 (also known as stromal cell-derived factor (SDF)-1α) is expressed at high levels in organs of metastatic destination such as lungs and bone marrow [5]. Disruption of the CXCL12-CXCR4 chemokine – receptor interaction is already being exploited clinically for mobilization of normal hematopoietic stem cells (HSC) from the bone marrow for apheresis and therapeutic use in high-dose chemotherapy regimens with autologous HSC support. Approved agent for this indication^1^ is the small-molecule CXCR4 receptor antagonist plerixafor (AMD3100; Mozobil®) [6], now investigated for HSC mobilization in patients with diverse malignancies, including children and adolescents.^2^ Plerixafor has also demonstrated safety and efficacy as an anti-cancer agent in mobilizing leukemic blasts from their bone marrow niche to overcome stroma-mediated drug resistance [7]. In solid cancers, microenvironment-derived CXCL12 has been shown to stimulate survival, growth, and migration of CXCR4-expressing cancer cells in a paracrine fashion [8, 9], to recruit (in an endocrine mechanism) endothelial progenitor cells to promote tumor vasculogenesis [10], and to direct circulating cancer cells to niches of high-level CXCL12 expression [5]. These cellular actions are driven by a multitude of CXCR4 downstream signaling cascades activated through receptor-coupled G-proteins (Gαi being a central component [11]) and non-G-protein mechanisms (reviewed in [12, 13]). It is therefore not surprising that divergent signaling and cellular responses are being observed, among different cancers but also within a particular cancer type and uniform CXCR4 expression [11, 14, 15]. Hence, many disease-specific aspects of the CXCL12-CXCR4 cancer cell − microenvironment interaction and its molecular signaling events remain undefined, especially in rare cancers. Still, given promising anti-tumor activities in vitro and in animal tumor models in vivo [1, 2, 5, 10, 12, 15], compounds that target this interaction (foremost plerixafor) are in clinical investigation for the treatment of patients with diverse solid malignancies [13],^3^ thus raising interest in the role of CXCR4 signaling in rare cancers such as Ewing sarcoma.

Ewing sarcoma is an aggressive cancer of bone and soft tissues and the second most common bone sarcoma in children and young adults. Prognosis of metastatic or relapsed disease remains poor [16, 17] despite intensive multimodal therapies, and novel strategies that target molecular mechanisms of metastasis are being sought. Recent gene expression analyses revealed a correlation between Ewing sarcoma *CXCR4* expression and metastatic phenotype and shortened patient survival [18, 19]. This, together with metastatic predilection sites in *CXCL12* high-level organs [16], suggests the CXCL12-CXCR4 chemokine – receptor interaction as a potential therapeutic target in this cancer. Indeed, Krook et al. [20] showed that a highly dynamic up-regulation of *CXCR4* in response to environmental stresses increased the pro-metastatic migration and invasion capacities of Ewing sarcoma cell lines. In contrast, our previous study of CXCR4 protein expression in Ewing sarcomas revealed a correlation with tumor volume and suggested a role for CXCR4 in proliferation and tumor growth rather than metastasis [21]. Nevertheless, both studies indicated potential for plerixafor to disrupt respective pro-tumorigenic CXCR4 actions in Ewing sarcoma. Given this initial evidence for CXCR4 as a molecular target, matched with plerixafor as a targeted agent that reached clinical application in children, we aimed to investigate the anti-tumor activities of plerixafor in Ewing sarcoma. However, an unexpected increase in relative viability of Ewing sarcoma cell lines in vitro led us to primarily focus on the mechanisms underlying this observation.

## Methods

### Cell lines

Ewing sarcoma cell lines A673, TC-32, and TC-71 were originally received from the cell line bank at Children’s Hospital Los Angeles; CADO-ES1 from DSMZ (Braunschweig, Germany); and VH-64 from F van Valen (Institute of Experimental Musculoskeletal Medicine, University Hospital Münster). The low-passage cell culture DC-ES-6 was established in our laboratory and previously described [22]. LAN-5 neuroblastoma cells were originally provided by R Seeger (Los Angeles, CA) and HL-60 acute myeloid leukemia cells were purchased from ATCC (Manassas, VA). Short tandem repeat profiling was performed to verify cell line identities and all cells were tested to be free of mycoplasma. Cells were cultured in collagen-coated tissue culture flasks (CADO-ES1, DC-ES-6, VH-64) or uncoated flasks (all other cell lines) in RPMI 1640 medium with 10% fetal bovine serum (FBS) (both Invitrogen, Carlsbad, CA) at 37 °C and with 5% CO_2_.

### Compounds and reagents

Plerixafor (AMD3100) and dasatinib were from SelleckChem (Houston, TX), recombinant CXCL12 (SDF-1α) from R&D Systems (Minneapolis, MN), pertussis toxin (PTX) from Sigma Aldrich (St. Louis, MO), and granulocyte-colony stimulating factor (GCSF; Filgrastim) from Amgen (Breda, Netherlands). Cell proliferation and viability was measured using the WST-1 colorimetric assay according to manufacturer’s recommendations (Roche Applied Science, Penzberg, Germany).

### Migration and wound healing assays

Cells were starved in serum-free medium for 12 h before 6 × 10^4^ cells were seeded into ThinCert™ cell culture inserts (8 μm pores; Greiner Bio-One, Frickenhausen, Germany) and chemoattractants were added to wells of a 24-well plate. After 48 h, cells remaining on the ThinCert™ membrane upper surface were removed with a cotton tip and migrated cells were fixed in 4% paraformaldehyde for 10 min. Membranes were washed in phosphate buffered saline (PBS) and stained with 4′,6-diamidino-2-phenylindole (DAPI) for 10 min. Membranes were mounted onto microscopy slides and migrated cells were counted in 5 fields per membrane at 100× magnification. For wound healing, A673 cells were seeded onto collagen coated tissue culture plates. At 80% confluence, plerixafor was added as indicated to cell culture medium containing 10% FBS. After 12 h, a wound was created using a pipette tip. Cell debris was removed by washing with PBS and cell culture medium and plerixafor were added as before. Images were acquired at indicated time points and wound areas were quantified using Image J software and the MRI Wound Healing Tool plug-in (http://dev.mri.cnrs.fr/projects/imagej-macros/wiki/Wound_Healing_Tool).

### Flow cytometry

For cell cycle analysis, cells were cultured in standard growth medium containing 10% FBS. Cells were synchronized with 2 mM thymidine for 18 h, released into growth medium for 8 h, and synchronized again for 18 h before being released in growth medium containing plerixafor as indicated for another 72 h. 1 × 10^6^ cells were washed in PBS containing 0.2% albumin and 0.01% NaN3 and then fixed in 70% ethanol. 4 μl of RNAse A was added and 30 min later cell were stained with 2 μl of propidium iodine for 30 min. For analysis of CXCR4 expression, cells were grown to 70–80% confluence and 1 × 10^6^ cells were stained with 0.1 μg of phycoerythrin-cyanine 7-fluorochrome-conjugated CXCR4 antibody (clone 12G5; Cat-No. 25–9999-42) or IgG2aK isotype control (Cat-No. 25–4724-81; both eBioscience, Thermo Fisher Scientific, Waltham, MA) for 10 min at room temperature. Stained cells were analyzed on a FACS Canto II flow cytometer (BD Bioscience, Franklin Lakes, NJ) using FACS Diva and FlowJo v10 software (FlowJo LLC, Ashland, Oregon). Relative fluorescence intensity (RFI) was calculated as the median fluorescence intensity of cells stained with specific CXCR4 antibody relative to those stained with isotype control.

### Western blotting

Procedures and buffers were as previously described [23]. CXCR4 antibodies were from abcam (N-terminal: Cat-No. ab2074; C-terminal: ab13854; Cambridge, UK); phospho-AKT (Ser473) (Cat-No. 9271), phospho-ERK1/2 (Thr202/Tyr204) (Cat-No. 9102), phospho-JNK (Thr183/Tyr185) (Cat-No. 9521), phospho-RPS6 (Ser235/236) (Cat-No. 2215), phospho-SRC (Tyr416) (Cat-No. 2101), and phospho-PDGFRB (Tyr751) (Cat-No. 3161) were from Cell Signaling Technology (Beverly, MA); β-actin (Cat-No. sc-47,778) was from Santa Cruz Biotechnology (Santa Cruz, CA). Secondary horseradish-peroxidase-conjugated antibodies were from Cell Signaling (anti-mouse, Cat-No. 7076) and BD Pharmingen (anti-rabbit, Cat-No. 554021; Franklin Lakes, NJ).

### Phospho-receptor tyrosine kinase array

The Proteome Profiler™ Human Phospho-RTK Array kit (Cat-No. ARY001B; R&D Systems) was applied according to manufacturer’s instructions. In brief, membranes were incubated with 400 μg whole cell lysate overnight at 4 °C. After washing with provided buffers, membranes were incubated with supplied horseradish-peroxidase-conjugated pan-phospho-tyrosine antibody and visualized by chemiluminescence. Pixel densities of RTK capture spots and controls were analyzed using Image J software (version 1.49 s). Mean pixel densities of duplicate RTK spots were normalized to mean pixel densities of control spots of respective membranes. Fold changes and Z-scores were calculated based on these normalized mean pixel densities.

### siRNA transfection

Small interfering RNA (siRNA) directed at CXCR4 (FlexiTube SI02664242) and a non-silencing negative control (AllStars negative control; SI03650318) were functionally verified by the manufacturer (Qiagen; Hilden, Germany) and in prior publications [24, 25]. 3 × 10^5^ TC-32 cells were seeded into 6-well culture dishes and reverse transfected with 50 nM siRNA using 24 μl HiPerfect reagent (Qiagen) in 2.3 ml standard growth medium according to the manufacturer’s protocol.

### Real-time quantitative reverse transcription PCR

RNA was isolated using the GeneJET RNA Purification Kit (Thermo Fisher Scientific). 2 μg of RNA was reverse transcribed with random hexamers and M-MLV reverse transcriptase (Promega, Madison, WI). PCR reactions of 12 μl contained 2 μl cDNA, 5.5 μl SYBR Green PCR mix (Applied Biosystems, Foster City, CA), and 0.5 μM primers (CXCR4: forward 5’-GAGGAAATGGGCTCAGGG, reverse 5’-AGTCAGCAGGAGGGCAGGG; GAPDH: forward: 5’-GAAGGTGAAGGTCGGAGTC, reverse: 5’-GAAGATGGTGATGGGATTTC). Amplification was performed in duplicate reactions at 50 °C for 2 min, followed by 95 °C for 10 min and 40 cycles of 95 °C (15 s) and 60 °C (1 min) on a Fast Real-Time PCR System (Applied Biosystems). Complementary DNA concentrations were adjusted to Ct (theshold cycle) values of GAPDH control gene to ensure equal amplification efficiencies. Gene expression was calculated by ΔΔCt-method relative to GAPDH.

### Statistics

Statistical significance was calculated using ANOVA for all analyses of multiple different conditions, with Sidak correction for post-hoc pairwise comparisons. Independet pairwise comparisons were calculated using t-test, and dependent pairwise comparisons were calculated using t-test with Benjamini-Hochberg (FDR) correction. Calculations were performed in Microsoft Excel and GraphPad Prism 6.0 software. Significance is indicated as *p* < 0.05 (*), *p* < 0.01 (**) and *p* < 0.001 (***), while ns indicates non-significant *p*-value.

## Results

### Plerixafor promotes proliferation of Ewing sarcoma cell lines in vitro

**Fig. 1:**
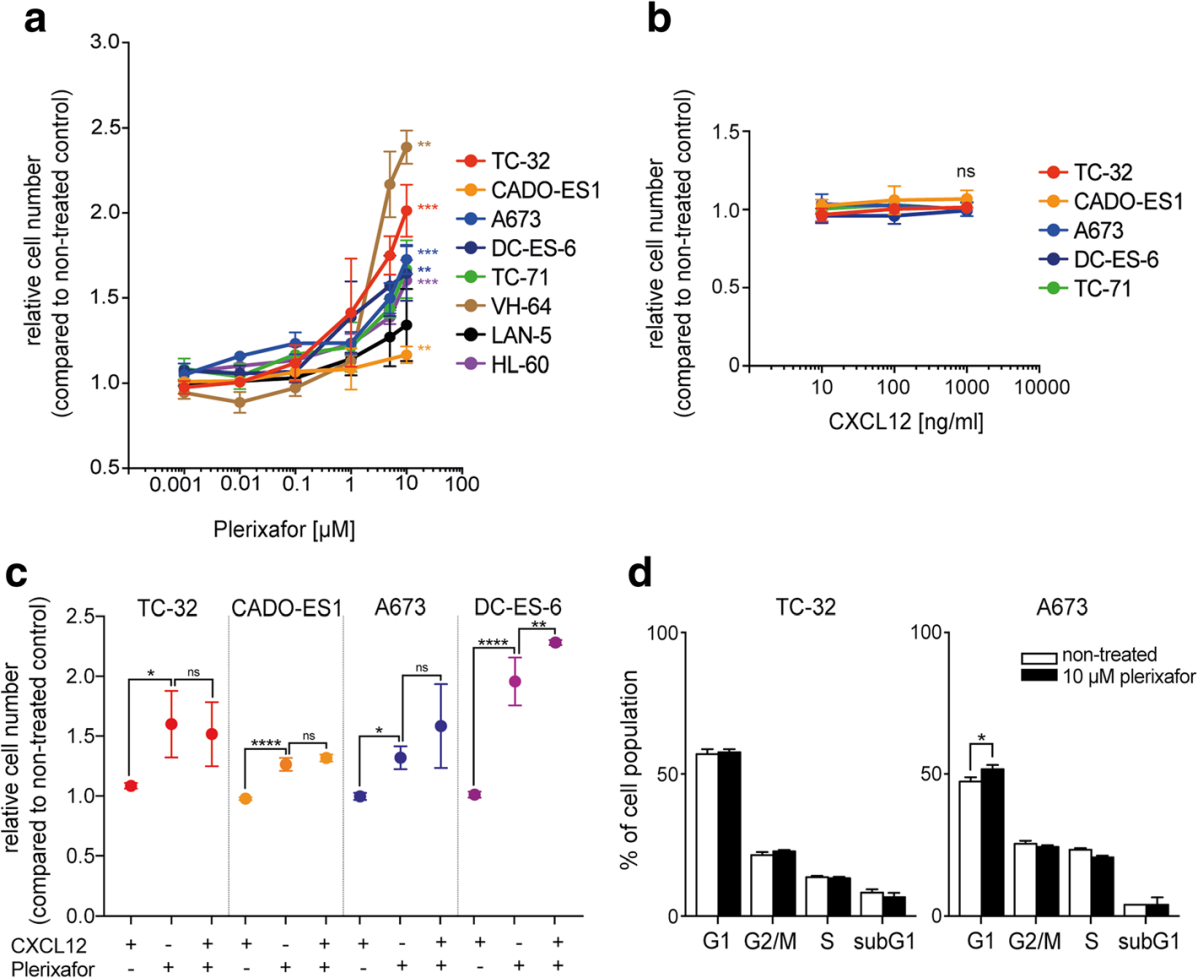
Plerixafor (AMD3100) but not CXCL12 promotes proliferation of Ewing sarcoma cell lines in vitro. **a** Plerixafor induces a dose-dependent increase in relative cell number. Cells were cultured in standard growth conditions (10% serum) and treated as indicated for 72 h before analysis of relative cell viability and proliferation by WST-1 colorimetric assay. Statistical significance was calculated for pairwise comparisons of relative cell number at 10 μM of plerixafor and respective non-treated control. **b** CXCL12 (SDF-1α) ligand alone does not significantly affect relative cell numbers. **c** CXCL12 (100 ng/ml) does not alter proliferative effects of plerixafor (10 μM). Assays were performed as in (**a**). Graphs (**a**-**c**) represent the mean ± standard deviation (SD) of at least three independent experiments. **d** Plerixafor-associated increase in relative cell numbers is not due to apoptosis of non-treated control cells. Cells grown in standard conditions were synchronized and treated for 72 h before analysis of DNA content by propidium iodine flow cytometry. Graphs represent the mean of three experiments

To investigate plerixafor (AMD3100) as an inhibitor of proliferative CXCR4 signaling in Ewing sarcoma, we performed in vitro cell proliferation and viability assays in several Ewing sarcoma cell lines including the low-passage cell culture DC-ES-6*.* Cells were exposed to plerixafor in standard culture conditions in the presence of 10% serum for 72 h. A dose range of 1 nM to 10 μM was assessed, covering clinical peak plasma concentrations of ~ 1 μM [26]. Unexpectedly, plerixafor induced a dose-dependent increase in relative cell numbers in all cell lines, reaching 1.4 fold at 1 μM in TC-32 and DC-ES-6 cells and up to 2.4 fold at 10 μM in VH-64 cells (Fig. 1a). A similar principal effect was observed for LAN-5 neuroblastoma and HL-60 acute myeloid leukemia cells that were included as controls with well-documented CXCR4 surface expression [27, 28]. In contrast, CXCL12 (SDF-1α) ligand did not affect Ewing sarcoma cell proliferation as a single agent (Fig. 1b) or alter the proliferative effects of plerixafor (Fig. 1c). Granulocyte-colony stimulating factor, the first-line agent in hematopoietic stem cell mobilization, and plerixafor-vehicle DMSO also showed no effects on relative cell numbers (Additional file 1). Cell cycle analysis was performed to exclude a relative increase in apoptosis of non-treated cells as a potential bias in this cell proliferation and viability assay (Fig. 1d). With 72 h of plerixafor treatment, this did not reveal an increase in mitotic cell populations in response to plerixafor.

### Ewing sarcoma cell lines show chemotactic migration to plerixafor

**Fig. 2:**
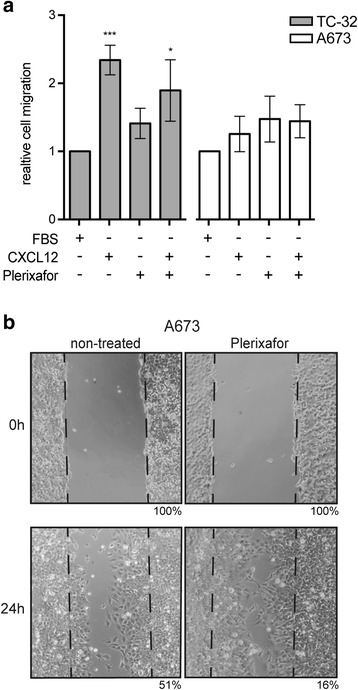
Ewing sarcoma cell lines show chemotactic migration to plerixafor. **a** TC-32 and A673 cells were cultured in serum-free medium and migration to FBS (10%), CXCL12 (100 ng/ml) and plerixafor (1 μM) was analyzed after 48 h. Graphs represent means ± SD of three independent experiments performed in triplicates. **b** Plerixafor (0.1 μM) promotes wound healing in a confluent A673 monolayer grown in standard conditions, documented by bright field microscopy at 40× magnification. Numbers indicate percent wound gap as quantified by Image J software and MRI Wound Healing Tool. Photographs are representative of three independent experiments

Given the well-documented role of CXCL12-CXCR4 signaling in cancer cell migration, we evaluated whether plerixafor stimulated not only proliferation but also migration of Ewing sarcoma cells in a “ligand-like” manner. Indeed, TC-32 cells showed chemotactic migration towards plerixafor, although this effect remained inferior to CXCL12, which served as positive control (Fig. 2a). In A673 cells in contrast, plerixafor-directed migration was increased compared to CXCL12, though both did not reach significance. Furthermore, plerixafor appeared to accelerate wound closure in a wound-healing assay (Fig. 2b). Together these findings indicate an unexpected potential for plerixafor to stimulate proliferation and chemotactic migration of Ewing sarcoma cells in vitro.

### Cell lines group into CXCR4-high and -low surface expression

**Fig. 3:**
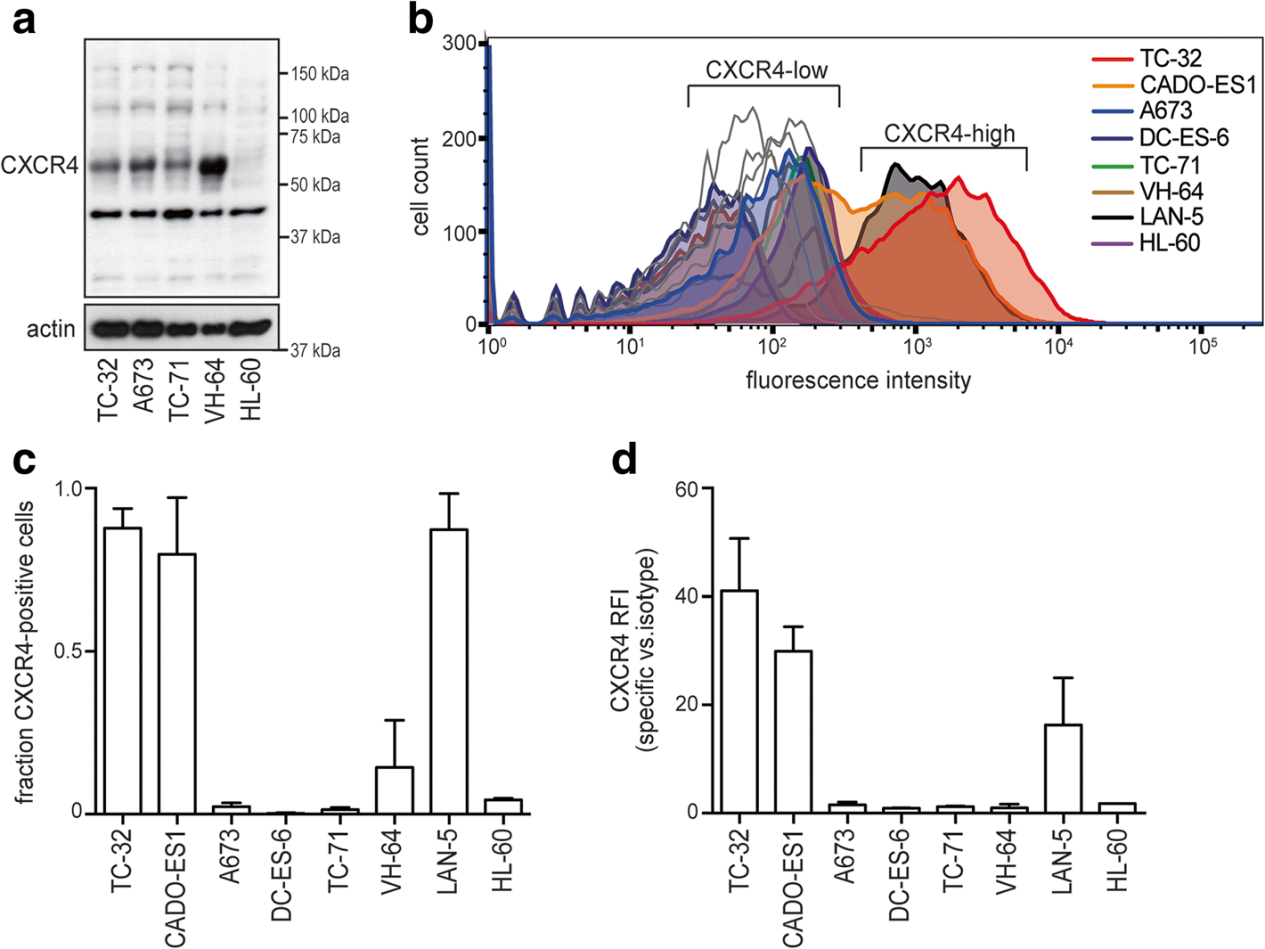
Ewing sarcoma cell lines group into CXCR4-high and -low surface expression. **a** Western blot analysis of total CXCR4 protein reveals multiple isoforms. Cells were grown to 70–80% confluence in standard conditions. Actin served as loading control. (**b**)-(**d**) CXCR4 surface expression distinguishes CXCR4-high and -low cell lines. Cells were grown as to 70–80% confluence in standard conditions and analyzed for CXCR4 surface expression by flow cytometry. LAN-5 neuroblastoma cells served as CXCR4-high positive control. **b** Representative flow cytometry plots; open graphs represent isotype-antibody controls. **c** CXCR4 positive cell populations depicted as mean ± SD of three independent analyses. **d** Relative fluorescence intensities of CXCR4 positive cell populations in (**c**). Corresponding data in serum-free conditions are provided in Additional file 1

We next investigated how these plerixafor effects related to CXCR4 receptor expressions of our cell line panel. Splicing isoforms and post-translational modifications such as glycosylation and ubiquitination can affect CXCR4 surface expression and function [27, 29]. Because such *CXCR4* splicing variants have been reported in Ewing sarcoma [30, 31], we first examined CXCR4 protein expression in Western blots of whole cell lysates. This revealed multiple bands as previously shown in other cancers [27, 29] (Fig. 3a). A band of ~ 45 kDa corresponded to the glycosylated CXCR4 monomer and was present in all cell lines including HL-60, whereas a 55 kDa isoform was restricted to Ewing sarcoma lines and thus dispensable for the proliferative plerixafor-response of HL-60 cells. Using an alternative CXCR4 antibody, we detected additional bands, also attributable to previously described isoforms and suggestive of post-translational modifications (Additional file 1). However, neither isoform correlated with CXCR4 surface expression, as in addition to HL-60, four Ewing sarcoma cell lines (A673, TC-71, VH-64, DC-ES-6) showed minimal detectable surface expression (Fig. 3b and c). In contrast, TC-32 and CADO-ES1 cell lines revealed surface expression in more than 75% of their population (Fig. 3c), at levels (assessed by relative fluorescence intensity, RFI) that exceeded those of LAN-5, classified as high-level surface expressing among neuroblastoma cell lines [27] (Fig. 3d). Because CXCR4 expression of Ewing sarcoma cell lines had been found heterogeneous within cell populations and highly dynamic in response to serum deprivation and space constraints [20], we performed our analyses at a constant confluence of 70–80% and in both standard (10% serum) (Fig. 3) and serum-free growth conditions (Additional file 1). Cell lines with substantial CXCR4 surface expression (TC-32, CADO-ES1, LAN-5) recapitulated this heterogeneity in surface expression levels (Fig. 3b), and TC-32 and HL-60 cells demonstrated a RFI increase in serum-free medium, while the other cell lines did not show significant changes in surface-expressing fractions or RFI (Additional file 1). Thus, Ewing sarcoma cell lines consistently grouped into CXCR4-high (TC-32 and CADO-ES1) and CXCR4-low (A673, TC-71, VH-64, DC-ES-6) surface expression; indicating that substantial CXCR4 surface expression was not essential for proliferative and migratory responses to plerixafor that were observed in both groups.

### Plerixafor-induced proliferation does not require substantial CXCR4 expression but is associated with AKT activation

**Fig. 4:**
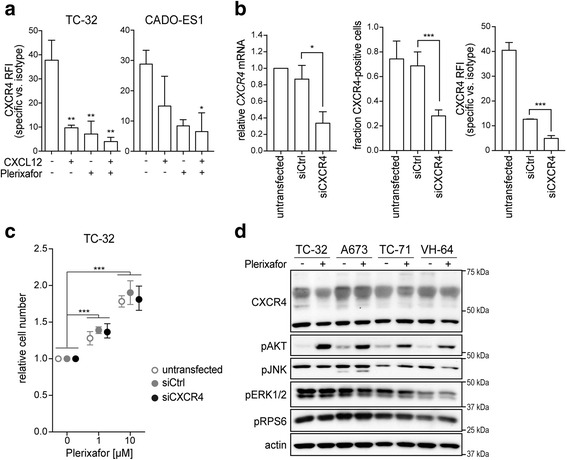
Plerixafor-induced proliferation does not require substantial CXCR4 surface expression but is associated with AKT activation. **a** CXCL12 and plerixafor bind to the CXCR4 receptor. TC-32 and CADO-ES1 cells were grown in standard conditions and treated with CXCL12 (100 ng/ml) and/or plerixafor (1 μM) for 12 h before flow cytometric analysis of CXCR4 signal with a 12G5-CXCR4 antibody. **b** Significant reduction in *CXCR4* mRNA (left panel) and surface protein expression (middle and right panel). Cells were transfected with CXCR4-targeting siRNA (siCXCR4) or non-silencing control (siCtrl) and analyzed by real-time quantitative PCR and flow cytometry after 48 h. **c** Proliferative plerixafor effects are maintained despite significant reduction in CXCR4 surface expression. 48 h after siRNA transfection, cells from (**b**) were treated with plerixafor for another 72 h. Relative cell number was measured by WST-1 assay. All graphs of this figure represent means ± SD of three independent experiments. **d** AKT is activated in response to plerixafor in CXCR4-high and -low cell lines. Cells grown in standard conditions were treated with plerixafor (1 μM) for 12 h or remained untreated. Whole cell lysates were analyzed by Western blotting. Actin was loading control

Even though substantial CXCR4 surface expression was dispensable for proliferative plerixafor effects in CXCR4-low cells, thereby suggesting a CXCR4 independent mechanism, we wished to determine whether plerixafor would bind and act through this receptor in CXCR4-high cells; also in view of a mechanistic study describing a weak CXCR4 agonism of plerixafor [32]. In CXCR4-high TC-32 and CADO-ES1 cells, CXCL12 ligand (as positive control) led to a decrease in CXCR4-RFI in flow cytometry (Fig. 4a), indicating functional ligand binding with CXCR4 receptor internalization [33, 34]. Plerixafor, which blocks binding of 12G5-flow cytometry antibody to the CXCR4 epitope [33, 34], resulted in a similar decrease in RFI, indicating that plerixafor bound CXCR4 to a similar extent. However, despite this demonstration of target binding, a significant decrease in CXCR4 surface expression due to siRNA silencing did not abrogate proliferative plerixafor effects in CXCR4-high cells (Fig. 4b and c). Also, plerixafor-binding to CXCR4 did not alter total CXCR4 expression (confirmed by densitometry in three independent experiments) or phosphorylation states of exemplary downstream signaling elements JNK, ERK1/2, and RPS6, involved in CXCR4-mediated migration and proliferation signaling [12, 35] (Fig. 4d). In contrast, AKT was phosphorylated upon plerixafor in this 12 h treatment course, both in CXCR4-high and -low cell lines (Fig. 4d).

### Plerixafor induces phosphorylation of receptor tyrosine kinases

**Fig. 5:**
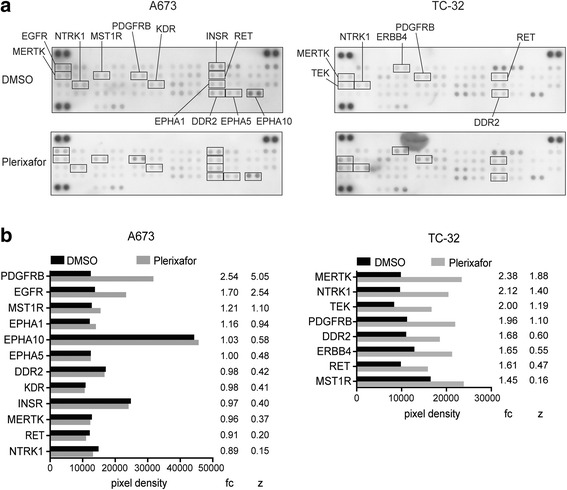
Plerixafor induces phosphorylation of receptor tyrosine kinases. **a** Phospho-RTK arrays were probed with whole cell lysates of CXCR4-low A673 and CXCR4-high TC-32 cells that were treated with 1 μM plerixafor or DMSO vehicle for 1 h. **b** RTKs activated by plerixafor. Mean pixel densities of duplicate RTK capture spots were measured and normalized to mean pixel densities of control spots of respective membranes. RTKs phosphorylated in the presence of plerixafor (mean normalized pixel density above the mean of all RTKs) were defined as active and considered for analysis. Fold changes (fc) of normalized mean pixel densities in plerixafor-treated (grey bars) compared to control cells (black bars) were calculated and RTKs with an activation fold change greater than the mean (Z-score (z) of fold change > 0) were defined as plerixafor-activated. The full data set is available in Additional file 2

The AKT kinase is a common signaling hub and feedback regulator of survival signaling through both G-protein coupled receptors and receptor tyrosine kinases (RTKs). Its activation following plerixafor treatment in both CXCR4-high and -low cell lines (Fig. 4d) prompted us to investigate RTKs as potential mediators of plerixafor-induced proliferation. We utilized an array of 49 RTKs to screen their phosphorylation patterns in a CXCR4-high (TC-32) and CXCR4-low (A673) cell line (Fig. 5a). Short-term (1 h) plerixafor treatment induced both increases and losses in RTK phosphorylation (full data set provided in Additional file 2). For potential targets of plerixafor signaling, we filtered for RTKs active in its presence, defined as a phosphorylation level above the mean of all RTKs. Candidates in which this phosphorylation represented a relative activation due to plerixafor as compared to the mean change in RTK phosphorylation (Z-score of the fold change of treated compared to non-treated cells > 0), were considered as plerixafor-activated (Fig. 5b). According to these criteria, DDR2, MERTK, MST1R, NTRK1, PDGFRB, and RET were activated in response to plerixafor in both CXCR4-high and -low cells. We therefore hypothesized that activation of RTKs contributed to plerixafor-induced cell proliferation and migration in Ewing sarcoma cell lines.

### Plerixafor and CXCR4 signaling interact with PDGFRB

**Fig. 6:**
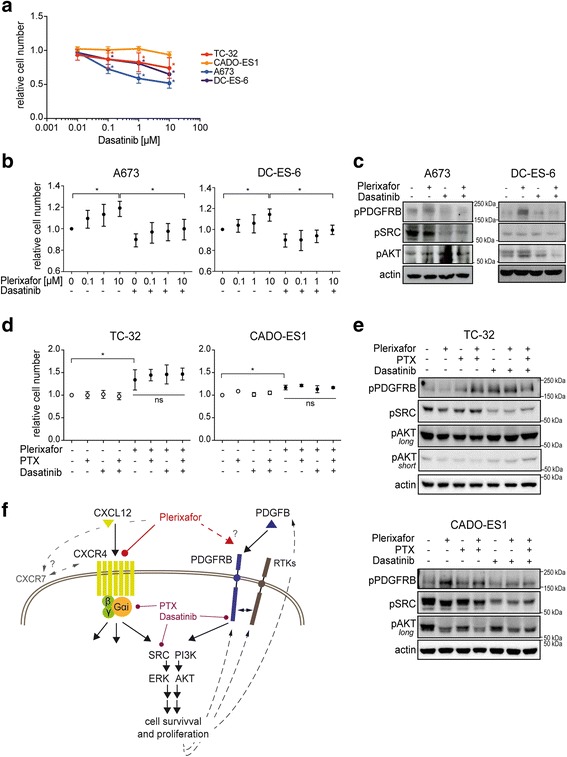
Plerixafor and CXCR4 signaling interact with PDGFRB. **a** Dasatinib dose-response of Ewing sarcoma cell lines. Cells were grown in standard conditions and treated for 72 h before relative cell numbers were measured by WST-1 colorimetric assay. **b** Dasatinib pre-treatment (100 nM) for 2 h reduces plerixafor-induced proliferation in A673 and DC-ES-6 CXCR4-low cells. Relative cell numbers were measured by WST-1 assay after 24 h. **c** Plerixafor induces phosphorylation of PDGFRB in CXCR4-low cells, which is abrogated by dasatinib pre-treatment. Cells were cultured in serum-free medium for 16 h, transferred to standard growth medium supplemented with 10% serum, and pre-treated with 100 nM dasatinib for 2 h. Plerixafor (1 μM) was added for another 1 h before preparation of whole cell lysates for Western blotting. Actin served as loading control. **d** Inhibition of Gαi-protein signal transduction does not abrogate plerixafor-induced proliferation in CXCR4-high cell lines or sensitize them to dasatinib. TC-32 and CADO-ES1 cells were pre-treated with pertussis toxin (PTX) (500 ng/ml) or dasatinib (100 nM) for 2 h before addition of plerixafor (10 μM) for another 72 h. Relative cell numbers were measured by WST-1 assay. All bar graphs in this figure depict mean ± SD of three independent experiments. **e** Both Plerixafor and PTX induce PDGFRB phosphorylation in CXCR4-high cells, which is not abrogated by dasatinib. Cells were cultured in serum-free medium for 16 h, transferred to standard growth medium, and pre-treated with PTX (500 ng/ml) and/or dasatinib (100 nM) for 2 h. Plerixafor (1 μM) was added for another 1 h before preparation of whole cell lysates for Western blotting. For phospho-AKT, an additional short exposure is shown for TC-32 cells. Actin served as loading control. All blots of this figure are representative of three independent experiments. **f** Graphical summary of our findings and hypothetical model of CXCR4 and RTK signaling crosstalk. Arrows indicate activation and blunt ends indicate inhibition

The platelet-derived growth factor receptor beta (PDGFRB) has been shown to contribute to Ewing sarcoma growth and metastasis in vitro and in vivo [36, 37] and demonstrated the most prominent activation upon plerixafor treatment in A673 CXCR4-low cells (Fig. 5b). To explore whether PDGFRB activation contributed to the proliferative effect of plerixafor, we employed the small-molecule inhibitor dasatinib. Dasatinib is a potent multi-tyrosine kinase inhibitor with high inhibitory activity on PDGFRB. Its broad target spectrum also comprises several RTKs and the non-receptor tyrosine kinase SRC [38], which has been reported as a CXCR4 signaling element downstream of the receptor-coupled Gαi protein [12, 39, 40]. To select an appropriate dose of dasatinib for our assays, dasatinib IC_50_ values (half maximal inhibitory concentration) for PDGFRB inhibition in Ewing sarcoma cell lines have not been reported, but respective IC_50_ values for SRC reached up to 50 fold above references [38, 41], implying relative resistance. Therefore, we extrapolated from a PDGFRB reference IC_50_ of 4 nM [38] and established dasatinib dose-responses of our cell lines (Fig. 6a), before selecting a dose of 100 nM for subsequent assays.

Indeed, pre-treatment of CXCR4-low cell lines A673 and DC-ES-6 with 100 nM dasatinib attenuated the proliferative effects of plerixafor (Fig. 6b) and plerixafor-induced phosphorylation of PDGFRB was prevented (Fig. 6c). In contrast and as expected for CXCR4-low cells, the CXCR4 downstream kinase SRC was not affected upon plerixafor treatment, and dasatinib abrogated baseline SRC phosphorylation in A673 but not DC-ES-6 cells (Fig. 6c), attributing its effects on plerixafor-mediated cell proliferation to an inhibition of PDGFRB rather than its co-target SRC.

In contrast to CXCR4-low cell lines, dasatinib did not abrogate proliferative plerixafor responses in CXCR4-high TC-32 and CADO-ES1 cells (though the overall plerixafor response of CADO-ES1 remained limited) (Fig. 6d). To test whether their high-level CXCR4 surface expression with potential for compensatory signaling contributed to this resistance, we utilized pertussis toxin (PTX), a potent non-specific inhibitor of Gαi-proteins that blocks Gαi signal transduction of plerixafor and CXCR4 [39, 40, 42]. However, pertussis toxin did not prevent plerixafor-induced proliferation of TC-32 or CADO-ES1 cells, either alone or in combination with dasatinib (Fig. 6d). Furthermore, pertussis toxin did not abrogate plerixafor-induced PDGFRB phosphorylation in CADO-ES1 cells. In TC32 cells, effects of plerixafor alone on PDGFRB phosphorylation even remained variable throughout independent experiments but were more prominent with combined plerixafor- and pertussis toxin-mediated inhibition of CXCR4 signaling (Fig. 6e, best representative of three independent experiments); indicating that inhibition of CXCR4 signaling triggered activation of PDGFRB. In keeping, and in contrast to A673 and DC-ES-6 cell lines, dasatinib treatment of TC-32 and CADO-ES1 resulted in a profound inhibition of SRC kinase in both lines but with sustained PDGFRB activation at baseline (CADO-ES1) or even increased levels (TC-32) (Fig. 6e), supporting that PDGFRB activation in CXCR4-high TC-32 and CADO-ES1 cells is in feedback response to losses of CXCR4, Gαi, or SRC signaling input. Interestingly, in response to plerixafor and/or pertussis toxin, phosphorylation of SRC and AKT remained unchanged or only slightly decreased (Fig. 6e), consistent with their role as downstream signaling and feedback nodes to both RTK and CXCR4 pathways. Of note, PDGFRB phosphorylation alone did not fully correlate with cellular proliferation responses (Fig. 6d and e), indicating that individual activities of feedback loops and of dasatinib at PDGFRB versus SRC kinases affect the net proliferative outcome for each CXCR4-high or -low cell line (Fig. 6f). In parallel, additional RTKs affected by plerixafor (Fig. 5b) point to a broader signaling interconnection between CXCR4 and receptor tyrosine kinases in Ewing sarcoma.

## Discussion

### Plerixafor can promote Ewing sarcoma cell proliferation

Our evaluation of plerixafor in Ewing sarcoma cell lines revealed unexpected pro-proliferative and chemotactic properties. Together with Berghuis and colleagues [21], we previously reported that plerixafor abrogated a CXCL12-induced increase in cell proliferation and viability, which we did not observe in this study. However, while based on a similar colorimetric cell proliferation and viability assay, those experimental conditions were distinct, in that a different panel of Ewing sarcoma cell lines was grown and treated in the absence of serum for seven days. Distinct serum and concomitant CXCL12 levels that alter baseline cell viabilities and activations of CXCR4 and growth factor receptors provide one possible explanation for our divergent findings. Indeed, Kim et al. [43] demonstrated that plerixafor protected multiple myeloma cell lines from apoptosis in a 3–5 day time course of serum-free culture, but resulted in unchanged relative viability compared to control cells at later time points. Therefore, we here excluded apoptosis as a confounding mechanism in our 3-day assays. Despite their heterogeneous findings, our previous [21] and current studies together endorse a role for CXCR4 signaling in Ewing sarcoma proliferation in vitro. Whereas our observation of a pro-proliferative capacity of plerixafor under certain conditions in vitro alone does not question its general anti-cancer potential, it calls for investigations into underlying mechanisms, beyond serum content as a (very limited) model of the chemokine and growth factor microenvironment in vivo.

### Cellular target receptors of pro-proliferative plerixafor actions

One central limitation to our study is that we did not yet investigate how plerixafor elicits pro-proliferative and migratory responses in CXCR4-low Ewing sarcoma cell lines. Our analyses of CXCR4 protein expression complement previously published RNA and flow cytometry data and provide a first overview of protein isoforms, but did not discern functionally distinct patterns. Instead, flow cytometry of our cell line panel confirmed the heterogeneous and dynamic CXCR4 surface expression reported by Krook et al. [20]. While general classes of high- and low-level CXCR4 expression remained consistent, such broad expression ranges within one cell population may lead to an underestimation of actual surface CXCR4. Thus, in a favorable cellular background of RTK expressions and feedback activities, relative low-level, dynamic CXCR4 surface expression such as in CXCR4-low cells or following (subtotal) CXCR4 siRNA knockdown may suffice to provide the cellular target receptor for pro-proliferative plerixafor actions.

The chemokine receptor CXCR7 has been identified as a second CXCL12 receptor [44]. Reporting plerixafor as an allosteric agonist at CXCR7, Kalatskaya and colleagues [45] introduced CXCR7 as a potential alternative mediator of pro-proliferative plerixafor signaling. While providing another hypothesis for plerixafor actions in CXCR4-low cells, data so far on CXCR7 are limited [46]. Although several reports involve CXCR7 in the progression (though not necessarily proliferation) of cancers [44, 47], others describe anti-tumorigenic function [48]. Moreover, exact CXCR7 signaling and non-signaling events and the roles of co-internalization with CXCR4 and of its primary ligand CXCL11 (I-TAC, Interferon-inducible T cell chemoattractant) remain open [44, 46, 47]. In Ewing sarcoma, analyses of *CXCR7* expression revealed controversial results with positive [30] and negative [18, 19] correlations with patient survival. At the same time, analyses of CXCR7 protein expression in Ewing sarcoma cell lines (including CXCR4-low lines) point out very limited total protein [49] and surface [21] expressions compared to CXCR4, suggesting that our findings in both CXCR4-high and -low cell lines are not primarily mediated by CXCR7; which nevertheless will be an important area of study in this context.

Of note, a third hypothesis of the small-molecule inhibitor plerixafor acting through cellular target receptors other than CXC chemokine receptors has not been addressed. While concrete evidence from ours or other studies is lacking, this possibility remains open.

### Transduction of pro-proliferative plerixafor signals

When acting through the CXCR4 receptor in CXCR4-high (or even CXCR4-low) cells, a weak agonistic activity of plerixafor at CXCR4 [32] provides an intriguing potential mechanism for the observed (counterintuitive) pro-proliferative effect. However, other studies dispute this one mechanistic report [6, 33]. Proliferative responses despite a significant reduction in surface CXCR4 expression (siRNA knockdown) or blockage of Gαi-protein signal transduction also argue against this mechanism, but do not altogether exclude it. Moreover, we did not profile the full multitude of CXCR4 downstream signaling cascades for activating plerixafor agonism. Yet, SRC and AKT did not suggest agonistic activation of the CXCR4 pathway in short-term treatment. Because we focused on plerixafor as the one CXCR4 inhibitor in clinical application in pediatric patients, we furthermore did not yet investigate whether compounds of distinct structure and purely antagonistic action at CXCR4 would elicit similar proliferative and migratory responses in CXCR4-high and -low Ewing sarcoma cell lines. Thus although remaining an important aspect, our results as yet do not support a mechanism of weak agonistic activity at CXCR4 for plerixafor-associated cell survival and proliferation. At the same time, with sustained or no more than slightly decreased activity of downstream elements SRC and AKT, plerixafor and pertussis toxin lack a positive control of their respective CXCR4 or Gαi inhibition. While SRC and AKT signaling positions downstream of both CXCR4 and PDGFRB pathways and within the proposed feedback loop provide a plausible explanation for their sustained activities, future studies should include independent controls when using pertussis toxin to dissect Gαi dependent and independent pathways of plerixafor signaling.

### Feedback survival signaling as a novel mechanism of CXCR4 and RTK interaction

Plerixafor treatment of Ewing sarcoma cell lines led to changes in RTK phosphorylation patterns in both CXCR4-high and -low lines. Activation of the survival-signaling hub AKT with longer-term plerixafor treatment and the involvement of several RTKs favor an indirect mechanism aimed at maintaining net cellular proliferation and survival signaling. Supporting this hypothesis, not only plerixafor resulted in activation of PDGFRB but also inhibition of the CXCR4 signaling pathway on the level of Gαi (pertussis toxin) and in TC-32 cells SRC (dasatinib). Sustained activation of SRC and AKT, downstream elements to both CXCR4 and PDGFRB, corroborate a mechanism of feedback survival signaling through these signaling nodes. Individual cellular dynamics of these feedback regulations provide a plausible explanation for variable inhibitor responses of SRC and AKT observed between different cell lines. Thus, our findings provide first evidence for a signaling crosstalk between CXCR4 and RTKs with pro-tumorigenic potential in pediatric sarcomas. And while exact mechanisms remain undefined in this manuscript, our data indicate a feedback survival signaling interaction between CXCR and PDGFRB.

In carcinomas of the breast and prostate, interactions of CXCR4 with RTKs have previously been reported. The RTK ERBB2 was shown to promote CXCR4 expression and thereby metastasis [50], while in turn CXCL12 ligand transactivated ERBB2 [39]. This involved a CXCL12-CXCR4-Gαi-SRC pathway. However in contrast to Ewing sarcoma cell lines, RTK activations were here abrogated by plerixafor, pertussis toxin, and SRC inhibitors, indicating RTKs as downstream elements rather than compensatory signals [39, 40]. Of interest to future studies in sarcomas, silencing of CXCR4 axis contributions sufficed to compromise initial establishment of prostate cancers in bone microenvironment, whereas established bone tumors were sensitive only to RTK inhibitors [40].

Among the several plerixafor-activated RTKs in Ewing sarcoma cell lines, PDGFRB demonstrated most pronounced activation in phospho-tyrosine arrays. Long shown to be expressed and active in Ewing sarcoma, PDGFRB and autocrine and paracrine feedback loops with its ligand PDGFB (platelet-derived growth factor beta) contribute to sarcoma cell proliferation and migration [36, 37]. However, PDGFRB inhibitors showed no more than limited clinical activity [51], possibly because activating mutations are rare in Ewing sarcoma [37]. Interestingly, Hamdan and colleagues recently demonstrated that Ewing sarcoma cell lines up-regulated PDGFB ligand in response to stromal-derived CXCL12 in vitro and in vivo; and that PDGFB promoted maturation of bone marrow-derived pericyte progenitor cells and thereby tumor vasculogenesis; whereas plerixafor diminished PDGFB expression and resulted in compromised tumor vasculature and apoptosis in vivo [49, 52]. While these studies provide a hypothetical mechanistic background for CXCR4 – PDGFRB interactions in vivo, our data highlight that plerixafor not only affects Ewing sarcoma’s tumor cell – microenvironment interaction but can trigger cellular survival signaling cascades, both possibly amenable to tyrosine kinase co-targeting strategies. However, these hypotheses require further validation in vivo.

## Conclusions

The unexpected observation that plerixafor treatment resulted in an increase in relative cell number and migration of Ewing sarcoma cell lines in vitro raises a number of questions: Is this effect specific to cancer type and model, experimental conditions in vitro, and the CXCR4-inhibiting agent used? Is it mediated through CXCR4, in an agonistic and / or antagonistic manner, or does it involve CXCR7 or even distinct receptors? And what are the cellular signaling mechanisms involved? In this manuscript we began to address these questions and our data as yet support feedback survival signaling between the CXCR4 chemokine receptor and the growth factor and RTK network, in Ewing sarcoma PDGFRB in particular, as a plausible mechanism. The roles of low-level CXCR4 expression, isoform variants, and the CXCR7 chemokine receptor however remain open. In future studies, it will be important to define whether CXCR4 – RTK feedback among neoplastic cells and with their primary or metastatic stromal microenvironment can confer resistance to plerixafor anti-tumor activities in vivo, and whether such mechanisms apply to the frequent cancer types and are amenable to RTK co-targeting strategies; or whether our observations remain relevant to the interpretation of in vitro cell culture data only.

## Additional files

Additional file 1: **Figure S1.** Granulocyte-colony stimulating factor and DMSO vehicle do not induce proliferation of Ewing sarcoma cell lines in vitro*.* **Figure S2.** Serum-deprivation does not alter cell line groups of CXCR4-high and -low surface expressions. (PDF 625 kb)

Additional file 2: **Table S1.** Plerixafor treatment changes receptor tyrosine kinase phosphorylation patterns in CXCR4-high and -low Ewing sarcoma cell lines. (PDF 1503 kb)

## Abbreviations

AKT: protein kinase B
ATCC: American type culture collection
Ct: threshold cycle
CXCL11: C-X-C chemokine motif 11
CXCL12: C-X-C chemokine motif 12
CXCR4: C-X-C motif chemokine receptor 4
CXCR7: C-X-C motif chemokine receptor 7
DAPI: 4′,6-diamidino-2-phenylindole
DDR2: Discoidin domain-containing receptor 2
DMSO: Dimethyl sulfoxide
DSMZ: Deutsche Sammlung von Mikroorganismen und Zellkulturen
ERBB2: erb-b2 receptor tyrosine kinase 2
ERK1/2: extracellular regulated MAP kinase (p44/42MAPK)
FBS: fetal bovine serum
GAPDH: glyceraldehyde-3-phosphate dehydrogenase
GCSF: granulocyte-colony stimulating factor
HSC: hematopoietic stem cells
IC_50_: half maximal inhibitory concentration
JNK: mitogen-activated protein kinase 8 (MAPK8)
MERTK: c-mer proto-oncogene tyrosine kinase
M-MLV: moloney murine leukemia virus reverse transcriptase
MST1R: macrophage stimulating 1 receptor
NTRK1: neurotrophic receptor tyrosine kinase 1
PBS: phosphate buffered saline
PCR: polymerase chain reaction
PDGFRB: platelet-derived growth factor receptor beta
PTX: pertussis toxin
RET: ret proto-oncogene
RFI: relative fluorescence intensity
RPS6: ribosomal protein S6
RTK: receptor tyrosine kinase
SD: standard deviation
siRNA: small interfering RNA
SRC: SRC proto-oncogene, non-receptor tyrosine kinase

## Footnotes

1 http://www.ema.europa.eu/ema/index.jsp?curl=pages/medicines/human/medicines/001030/human_med_000910.jsp&mid=WC0b01ac058001d124; https://www.accessdata.fda.gov/scripts/cder/daf/index.cfm?event=overview.process&applno=022311. Accessed Sept 18, 2017.

2 https://www.clinicaltrials.gov/ct2/results?cond=Childhood+Cancer&term=Plerixafor&cntry1=&state1=&Search=Search. Accessed Oct 18, 2017.

3 https://www.clinicaltrials.gov/ct2/results?cond=Cancer&term=AMD3100&cntry1=&state1=&SearchAll=Search+all+studies&recrs=. Accessed July 11, 2017.
