# Supplementary material for: The CXCR4 antagonist plerixafor (AMD3100) promotes proliferation of Ewing sarcoma cell lines in vitro and activates receptor tyrosine kinase signaling
Source: Cell Commun Signal. 2018 May 18;16:21. doi: 10.1186/s12964-018-0233-2 (PMC5960216; doi:10.1186/s12964-018-0233-2)
Supplement: Supplementary file 1 — Figure S1. Granulocyte-colony stimulating factor and DMSO vehicle do not induce proliferation of Ewing sarcoma cell lines in vitro. Figure S2. Serum-deprivation does not alter cell line groups of CXCR4-high and -low surface expressions. (PDF 625 kb) [file 12964_2018_233_MOESM1_ESM.pdf]

Additional file 1: Supplementary Figures

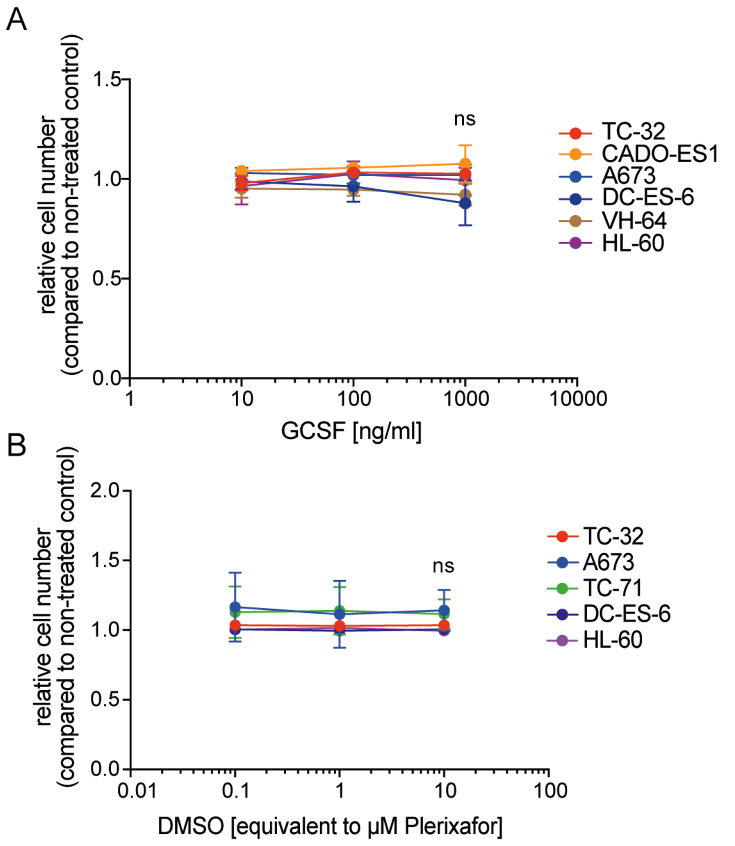

Fig S1. Granulocyte-colony stimulating factor and DMSO vehicle do not induce proliferation of Ewing sarcoma cell lines *in vitro*. (A-B) Ewing sarcoma cell lines and the low-passage cell culture DC-ES-6 were cultured in standard growth medium (10% serum) and treated for 72 h with (A) granulocyte-colony stimulating factor (GCSF) or (B) DMSO vehicle control. Relative cell numbers were measured by WST-1 colorimetric assay. Graphs represent mean ± standard deviation (SD) of three independent experiments. Statistical significance was calculated for pairwise comparisons of relative cell number at maximum doses of GCSF or DMSO and respective non-treated control; none reached significance.

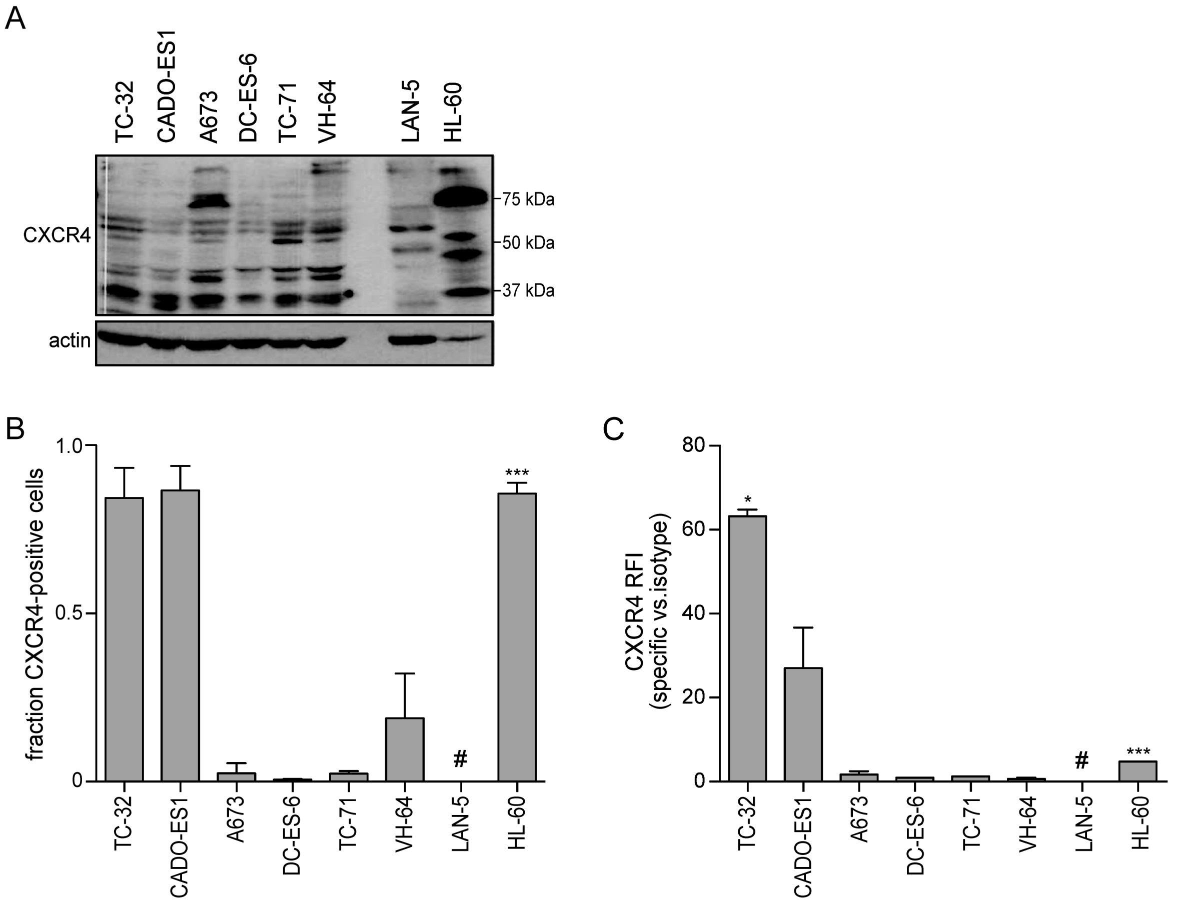

Fig S2. Serum-deprivation does not alter cell line groups of CXCR4-high and -low surface expressions. (A) Western blot analysis of total CXCR4 protein using an alternative (C-terminal) CXCR4 antibody reveals additional isoform bands. Whole cell lysates from Fig 3A were analyzed. Actin served as loading control. (B)-(C) Cells were grown to 70-80% confluence and starved in serum-free medium for 16 h before CXCR4 surface expression was analyzed by flow cytometry. LAN-5 neuroblastoma cells did not tolerate serum-free culture and were omitted from analysis; (B) depicts fractions of CXCR4-positive cells and (C) their relative fluorescence intensities (RFI; CXCR4 antibody relative to isotype control); asterisk (*) indicates significance of pairwise comparisons to corresponding culture in 10% serum (Fig 3).
