# Supplementary material for: The CXCR4 antagonist plerixafor (AMD3100) promotes proliferation of Ewing sarcoma cell lines in vitro and activates receptor tyrosine kinase signaling
Source: Cell Commun Signal. 2018 May 18;16:21. doi: 10.1186/s12964-018-0233-2 (PMC5960216; doi:10.1186/s12964-018-0233-2)
Supplement: Supplementary file 2 — Table S1. Plerixafor treatment changes receptor tyrosine kinase phosphorylation patterns in CXCR4-high and -low Ewing sarcoma cell lines. (PDF 1503 kb) [file 12964_2018_233_MOESM2_ESM.pdf]

Additional file 2: Supplementary Table

Table S1. Plerixafor treatment changes receptor tyrosine kinase phosphorylation patterns in CXCR4-high and -low Ewing sarcoma cell lines. This is the full data set corresponding to Fig 5. A phospho-RTK array was probed with whole cell lysates of CXCR4-low A673 and CXCR4-high TC-32 cells treated with 1 µM plerixafor or DMSO vehicle for 1 h. Mean pixel densities of duplicate RTK capture spots were measured and normalized to mean pixel densities of control spots of respective membranes. Fold changes (fc) of plerixafor-treated compared to control cells were calculated.

	A673			TC32		
	mean pixel density					
RTK	DMSO	Plerixafor	fc	DMSO	Plerixafor	fc
EGFR	13748.0	23305.5	1.70	2182.0	3928.5	1.80
ERBB2	13164.0	9942.5	0.76	14170.0	11516.5	0.81
ERBB3	3294.5	3405.0	1.03	3453.0	8607.5	2.49
ERBB4	9007.5	6901.5	0.77	12855.5	21262.0	1.65
FGFR1	8276.0	6360.0	0.77	4867.0	10510.0	2.16
FGFR2	8921.0	8665.5	0.97	6073.5	10274.0	1.69
FGFR3	7614.5	4650.0	0.61	5176.5	6715.0	1.30
FGFR4	9846.5	6375.0	0.65	6756.5	7459.5	1.10
INSR	24793.0	24092.0	0.97	50665.0	47081.0	0.93
IGF1R	26926.0	14093.5	0.52	36182.5	28787.0	0.80
AXL	4369.5	2920.5	0.67	1539.0	1659.0	1.08
TYR03	35164.5	23953.5	0.68	15339.0	10571.0	0.69
MERTK	12803.0	12310.5	0.96	9848.5	23400.0	2.38
MET	5569.0	3385.0	0.61	3960.5	8983.0	2.27
MST1R	12779.0	15454.0	1.21	16481.0	23866.5	1.45
PDGFRA	5712.5	4523.5	0.79	7391.0	9518.0	1.29
PDGFRB	12491.5	31714.0	2.54	11238.0	21971.0	1.96
KIT	9276.5	7679.0	0.83	14931.5	17872.5	1.20
FLT3	7788.5	4961.0	0.64	5947.0	8322.5	1.40
M-CSF R	15140.0	9776.0	0.65	14373.5	12339.5	0.86
RET	12167.0	11016.0	0.91	9849.0	15873.0	1.61
ROR1	9513.0	7374.5	0.78	2669.0	5206.5	1.95
ROR2	10366.5	8265.0	0.80	6728.0	4476.0	0.67
TIE1	5250.0	4613.5	0.88	3456.5	1665.0	0.48
TEK	10437.5	6740.5	0.65	8337.0	16702.0	2.00
NTRK1	14853.5	13164.0	0.89	9654.0	20442.0	2.12
NTRK2	5018.0	4295.0	0.86	4335.5	11669.5	2.69
NTRK3	9329.0	3853.0	0.41	6852.0	8847.0	1.29
VEGFRA	8758.5	8247.5	0.94	10988.0	9260.5	0.84
KDR	10847.0	10580.0	0.98	12564.0	15287.0	1.22
FLT4	20857.5	13783.0	0.66	19550.5	26369.5	1.35
MUSK	8262.0	5367.0	0.65	8137.0	11642.5	1.43
EPHA1	12138.5	14020.0	1.16	13374.0	13932.5	1.04
EPHA2	4902.5	4576.5	0.93	3678.0	5298.5	1.44
EPHA3	4086.5	1870.5	0.46	1736.0	1675.0	0.97
EPHA4	16254.0	12549.0	0.77	12279.5	6875.0	0.56
EPHA6	3633.0	2893.5	0.80	2970.5	4960.0	1.67
EPHA7	7360.5	4014.0	0.55	2887.5	4443.5	1.54
EPHB1	11317.5	6267.0	0.55	7159.0	9779.5	1.37
EPHB2	6605.0	4191.0	0.63	7281.0	9793.5	1.35
EPHB4	7299.5	4874.0	0.67	7012.0	5681.0	0.81
EPHB6	3966.5	2401.5	0.61	8088.0	5417.0	0.67
ALK	19251.0	16074.0	0.84	34938.5	30588.5	0.88
DDR1	13107.0	8960.5	0.68	16956.0	13041.0	0.77
DDR2	17087.0	16704.5	0.98	11030.0	18538.5	1.68
EPHA5	12479.5	12481.5	1.00	5875.5	8046.0	1.37
EPHA10	44229.0	45610.5	1.03	39823.0	34515.0	0.87
EPHB3	7405.0	5630.0	0.76	5630.5	8337.0	1.48
RYK	23198.0	22865.0	0.99	19124.5	37438.5	1.96
